# *N*-(2-Chloro­phen­yl)-1-phenyl­formamido 3-(2-nitro­phen­yl)propano­ate

**DOI:** 10.1107/S1600536812037981

**Published:** 2012-09-08

**Authors:** Hongxia Zhang, Donghui Qu, Jing Ma

**Affiliations:** a Gansu Health Center Hospital, Lanzhou 730000, Gansu Province, People’s Republic of China; b Institute of Medicinal Chemistry School of Pharmacy, Lanzhou University, Lanzhou 730000, Gansu Province, People’s Republic of China

## Abstract

In the title mol­ecule, C_22_H_17_ClN_2_O_5_, the nitro-substituted benzene ring makes a dihedral angle of 79.22 (1)° with the benzoyl ring and 53.03 (1)° with the chloro-substituted benzene ring. An intra­molecular C—H⋯O hydrogen bond occurs. The crystal structure features weak C—H⋯Cl and C—H⋯O inter­actions.

## Related literature

For applications of hydroxamic acid derivatives, see: Noh *et al.* (2009 ▶); Zeng *et al.* (2003 ▶). For the preparation, see: Ayyangark *et al.* (1986 ▶).
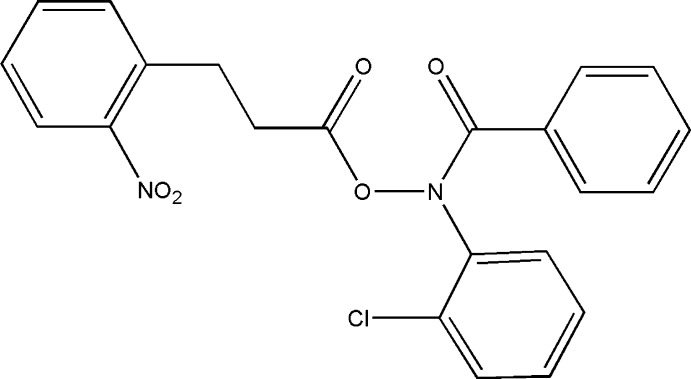


## Experimental

### 

#### Crystal data

C_22_H_17_ClN_2_O_5_
*M* *_r_* = 424.83
Monoclinic, 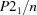

*a* = 14.698 (9) Å
*b* = 8.030 (5) Å
*c* = 17.495 (11) Å
β = 103.059 (7)°
*V* = 2011 (2) Å^3^
*Z* = 4
Mo *K*α radiation
μ = 0.23 mm^−1^
*T* = 296 K
0.26 × 0.23 × 0.22 mm

#### Data collection

Bruker APEXII CCD diffractometer
Absorption correction: multi-scan (*SADABS*; Sheldrick, 1996 ▶) *T*_min_ = 0.943, *T*_max_ = 0.952
8356 measured reflections
3697 independent reflections
2285 reflections with *I* > 2σ(*I*)
*R*_int_ = 0.045

#### Refinement

*R*[*F* ^2^ > 2σ(*F* ^2^)] = 0.047
*wR*(*F* ^2^) = 0.114
*S* = 1.02
3697 reflections
271 parameters
H-atom parameters constrained
Δρ_max_ = 0.18 e Å^−3^
Δρ_min_ = −0.26 e Å^−3^

### 

Data collection: *APEX2* (Bruker, 2009 ▶); cell refinement: *SAINT* (Bruker, 2009 ▶); data reduction: *SAINT*; program(s) used to solve structure: *SHELXS97* (Sheldrick, 2008 ▶); program(s) used to refine structure: *SHELXL97* (Sheldrick, 2008 ▶); molecular graphics: *SHELXTL* (Sheldrick, 2008 ▶); software used to prepare material for publication: *SHELXL97*.

## Supplementary Material

Crystal structure: contains datablock(s) global, I. DOI: 10.1107/S1600536812037981/zq2179sup1.cif

Structure factors: contains datablock(s) I. DOI: 10.1107/S1600536812037981/zq2179Isup2.hkl

Supplementary material file. DOI: 10.1107/S1600536812037981/zq2179Isup3.cml

Additional supplementary materials:  crystallographic information; 3D view; checkCIF report

## Figures and Tables

**Table 1:** Hydrogen-bond geometry (Å, °)

*D*—H⋯*A*	*D*—H	H⋯*A*	*D*⋯*A*	*D*—H⋯*A*
C8—H8*B*⋯Cl1^i^	0.97	2.68	3.627 (3)	167
C7—H7*A*⋯O1^ii^	0.97	2.60	3.520 (3)	158
C8—H8*A*⋯O2	0.97	2.47	3.092 (3)	121
C18—H18⋯O1^iii^	0.93	2.62	3.517 (4)	161
C4—H4⋯O1^iv^	0.93	2.64	3.468 (4)	149

Symmetry codes: (i) 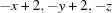; (ii) 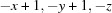; (iii) 
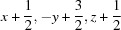
; (iv) 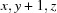.

**Fig. 1.**
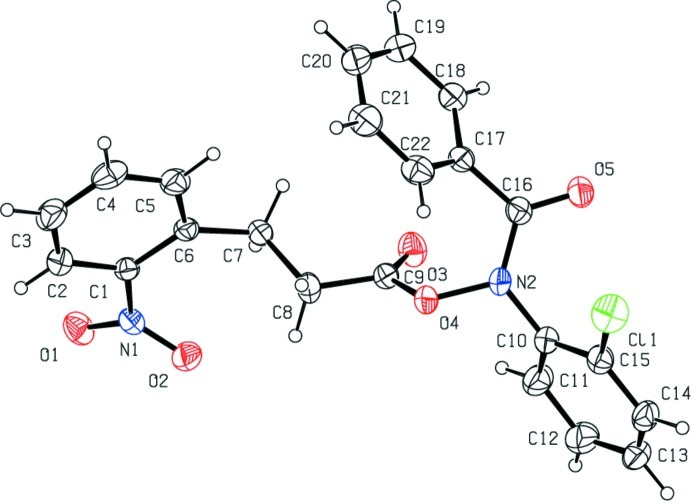
The molecular structure of the title compound with the atomic numbering and 30% probability displacement ellipsoids (H atoms are shown as small spheres of arbitrary radius).

## supplementary crystallographic information

### Comment

Hydroxamic acid derivatives have received considerable attention in recent years as the result of the discovery of their role in the biochemical toxicology of many drugs and other chemicals. Thus, these compounds continue to attract much attention as potential biological agents (Noh *et al.*, 2009; Zeng *et al.*, 2003).

The title compound, C_22_H_17_N_2_O_5_Cl, was prepared according to the method described by Ayyangark *et al.* (1986). The molecule contains three branched chains with its centre placed at midpoint of the N2 atom (Fig. 1). The phenyl ring C1—C6 makes dihedral angles of 79.22 (1)° with the phenyl ring C17—C22, and 53.03 (1)° with the phenyl ring C10—C15.

The crystal structure features weak C—H···Cl and C—H···O interactions (Table 1).

### Experimental

The title compound was prepared according to the method described by Ayyangark *et al.* (1986). The colourless crystals were grown from a solution of dichloromethane-methanol (1:3 *v*/*v*) by slow evaporation at room temperature.

### Refinement

All H atoms were positioned geometrically and allowed to ride on their parent atoms, with C—H = 0.93 Å and *U*_iso_ = 1.2*U*_eq_(C) for aromatic H atoms, and with C—H = 0.97 Å and *U*_iso_ = 1.2*U*_eq_(C) for methylene H atoms.

### Crystal data

**Table:**

C_22_H_17_ClN_2_O_5_	*F*(000) = 880
*M**_r_* = 424.83	*D*_x_ = 1.403 Mg m^−^^3^
Monoclinic, *P*2_1_/*n*	Mo *K*α radiation, λ = 0.71073 Å
Hall symbol: -P 2yn	Cell parameters from 1690 reflections
*a* = 14.698 (9) Å	θ = 2.4–22.2°
*b* = 8.030 (5) Å	µ = 0.23 mm^−^^1^
*c* = 17.495 (11) Å	*T* = 296 K
β = 103.059 (7)°	Block, colourless
*V* = 2011 (2) Å^3^	0.26 × 0.23 × 0.22 mm
*Z* = 4	

### Data collection

**Table:**

Bruker APEXII CCD diffractometer	3697 independent reflections
Radiation source: fine-focus sealed tube	2285 reflections with *I* > 2σ(*I*)
Graphite monochromator	*R*_int_ = 0.045
φ and ω scans	θ_max_ = 25.5°, θ_min_ = 2.4°
Absorption correction: multi-scan (*SADABS*; Sheldrick, 1996)	*h* = −17→13
*T*_min_ = 0.943, *T*_max_ = 0.952	*k* = −9→9
8356 measured reflections	*l* = −21→19

### Refinement

**Table:**

Refinement on *F*^2^	0 restraints
Least-squares matrix: full	H-atom parameters constrained
*R*[*F*^2^ > 2σ(*F*^2^)] = 0.047	*w* = 1/[σ^2^(*F*_o_^2^) + (0.0421*P*)^2^] where *P* = (*F*_o_^2^ + 2*F*_c_^2^)/3
*wR*(*F*^2^) = 0.114	(Δ/σ)_max_ = 0.001
*S* = 1.02	Δρ_max_ = 0.18 e Å^−^^3^
3697 reflections	Δρ_min_ = −0.26 e Å^−^^3^
271 parameters	

### Special details

**Table:**

Geometry. All e.s.d.'s (except the e.s.d. in the dihedral angle between two l.s. planes) are estimated using the full covariance matrix. The cell e.s.d.'s are taken into account individually in the estimation of e.s.d.'s in distances, angles and torsion angles; correlations between e.s.d.'s in cell parameters are only used when they are defined by crystal symmetry. An approximate (isotropic) treatment of cell e.s.d.'s is used for estimating e.s.d.'s involving l.s. planes.
Refinement. Refinement of *F*^2^ against ALL reflections. The weighted *R*-factor *wR* and goodness of fit *S* are based on *F*^2^, conventional *R*-factors *R* are based on *F*, with *F* set to zero for negative *F*^2^. The threshold expression of *F*^2^ > σ(*F*^2^) is used only for calculating *R*-factors(gt) *etc*. and is not relevant to the choice of reflections for refinement. *R*-factors based on *F*^2^ are statistically about twice as large as those based on *F*, and *R*- factors based on ALL data will be even larger.

### Fractional atomic coordinates and isotropic or equivalent isotropic displacement parameters (Å^2^)

**Table:**

	*x*	*y*	*z*	*U*_iso_*/*U*_eq_	
C1	0.54356 (14)	0.7597 (3)	−0.08505 (13)	0.0364 (5)	
C2	0.47799 (16)	0.8218 (3)	−0.14815 (14)	0.0499 (7)	
H2	0.4370	0.7502	−0.1809	0.060*	
C3	0.47420 (19)	0.9893 (4)	−0.16184 (17)	0.0625 (8)	
H3	0.4314	1.0326	−0.2047	0.075*	
C4	0.53416 (19)	1.0939 (4)	−0.11187 (18)	0.0626 (8)	
H4	0.5323	1.2081	−0.1210	0.075*	
C5	0.59688 (16)	1.0288 (3)	−0.04832 (16)	0.0525 (7)	
H5	0.6359	1.1014	−0.0145	0.063*	
C6	0.60438 (14)	0.8602 (3)	−0.03261 (13)	0.0375 (6)	
C7	0.67502 (14)	0.8013 (3)	0.03913 (13)	0.0441 (6)	
H7A	0.6520	0.7007	0.0590	0.053*	
H7B	0.6820	0.8856	0.0797	0.053*	
C8	0.77017 (14)	0.7660 (3)	0.02151 (13)	0.0457 (6)	
H8A	0.7677	0.6601	−0.0056	0.055*	
H8B	0.7841	0.8517	−0.0132	0.055*	
C9	0.84611 (15)	0.7607 (3)	0.09349 (15)	0.0417 (6)	
C10	1.07421 (15)	0.7137 (3)	0.15039 (14)	0.0456 (6)	
C11	1.05317 (18)	0.5495 (4)	0.15897 (17)	0.0646 (8)	
H11	0.9922	0.5189	0.1592	0.077*	
C12	1.1218 (2)	0.4293 (4)	0.16719 (17)	0.0701 (9)	
H12	1.1067	0.3178	0.1718	0.084*	
C13	1.21242 (19)	0.4745 (4)	0.16857 (16)	0.0627 (8)	
H13	1.2590	0.3938	0.1756	0.075*	
C14	1.23411 (17)	0.6372 (4)	0.15972 (14)	0.0567 (7)	
H14	1.2954	0.6674	0.1606	0.068*	
C15	1.16538 (16)	0.7572 (3)	0.14945 (13)	0.0467 (6)	
C16	0.99784 (16)	0.9673 (3)	0.18933 (15)	0.0428 (6)	
C17	0.92056 (15)	1.0900 (3)	0.16295 (14)	0.0414 (6)	
C18	0.86650 (17)	1.1325 (3)	0.21517 (15)	0.0527 (7)	
H18	0.8794	1.0861	0.2652	0.063*	
C19	0.79360 (18)	1.2432 (4)	0.19365 (18)	0.0616 (8)	
H19	0.7567	1.2696	0.2287	0.074*	
C20	0.7755 (2)	1.3147 (4)	0.12042 (19)	0.0677 (8)	
H20	0.7261	1.3891	0.1059	0.081*	
C21	0.8295 (2)	1.2771 (4)	0.06901 (18)	0.0666 (8)	
H21	0.8176	1.3274	0.0199	0.080*	
C22	0.90226 (18)	1.1643 (3)	0.08966 (16)	0.0558 (7)	
H22	0.9388	1.1385	0.0543	0.067*	
Cl1	1.19330 (5)	0.96079 (10)	0.13393 (4)	0.0716 (3)	
N1	0.54447 (14)	0.5788 (3)	−0.07543 (11)	0.0415 (5)	
N2	1.00361 (12)	0.8386 (3)	0.13960 (12)	0.0529 (6)	
O1	0.46940 (12)	0.5048 (2)	−0.09402 (10)	0.0596 (5)	
O2	0.61882 (12)	0.5081 (2)	−0.05065 (10)	0.0556 (5)	
O3	0.84162 (11)	0.7242 (2)	0.15847 (10)	0.0573 (5)	
O4	0.92840 (9)	0.8124 (2)	0.07409 (9)	0.0457 (4)	
O5	1.05461 (11)	0.9793 (2)	0.25171 (10)	0.0536 (5)	

### Atomic displacement parameters (Å^2^)

**Table:**

	*U*^11^	*U*^22^	*U*^33^	*U*^12^	*U*^13^	*U*^23^
C1	0.0354 (12)	0.0330 (15)	0.0410 (14)	0.0018 (10)	0.0094 (10)	−0.0036 (11)
C2	0.0460 (14)	0.0504 (19)	0.0493 (16)	0.0066 (12)	0.0021 (12)	−0.0035 (13)
C3	0.0597 (17)	0.063 (2)	0.0625 (19)	0.0194 (15)	0.0083 (14)	0.0084 (16)
C4	0.0661 (18)	0.0404 (17)	0.085 (2)	0.0127 (14)	0.0254 (17)	0.0051 (17)
C5	0.0465 (15)	0.0398 (17)	0.071 (2)	−0.0037 (12)	0.0134 (14)	−0.0118 (14)
C6	0.0331 (12)	0.0378 (16)	0.0429 (14)	−0.0005 (10)	0.0113 (11)	−0.0061 (12)
C7	0.0376 (13)	0.0526 (17)	0.0412 (14)	−0.0013 (11)	0.0068 (11)	−0.0110 (12)
C8	0.0360 (12)	0.0627 (18)	0.0366 (14)	−0.0044 (12)	0.0041 (10)	0.0003 (13)
C9	0.0373 (13)	0.0398 (16)	0.0467 (16)	−0.0008 (11)	0.0066 (11)	−0.0053 (13)
C10	0.0365 (13)	0.0526 (18)	0.0450 (15)	0.0034 (12)	0.0037 (11)	−0.0065 (13)
C11	0.0442 (15)	0.057 (2)	0.090 (2)	0.0011 (14)	0.0109 (15)	−0.0104 (17)
C12	0.068 (2)	0.052 (2)	0.087 (2)	0.0074 (16)	0.0099 (17)	−0.0058 (17)
C13	0.0543 (18)	0.074 (2)	0.0562 (18)	0.0248 (16)	0.0042 (13)	−0.0037 (16)
C14	0.0394 (14)	0.087 (2)	0.0448 (16)	0.0105 (15)	0.0123 (12)	−0.0005 (16)
C15	0.0445 (14)	0.0617 (19)	0.0346 (14)	0.0025 (13)	0.0103 (11)	0.0032 (13)
C16	0.0369 (13)	0.0468 (16)	0.0459 (15)	−0.0082 (11)	0.0119 (12)	−0.0032 (13)
C17	0.0410 (13)	0.0376 (15)	0.0448 (15)	−0.0030 (11)	0.0081 (11)	−0.0044 (12)
C18	0.0545 (15)	0.0526 (18)	0.0480 (16)	0.0045 (13)	0.0056 (13)	−0.0054 (14)
C19	0.0560 (16)	0.063 (2)	0.066 (2)	0.0100 (14)	0.0133 (15)	−0.0114 (17)
C20	0.0652 (18)	0.050 (2)	0.078 (2)	0.0126 (14)	−0.0057 (17)	−0.0105 (17)
C21	0.088 (2)	0.0488 (19)	0.0571 (19)	0.0096 (16)	0.0036 (17)	0.0065 (15)
C22	0.0701 (18)	0.0462 (18)	0.0515 (17)	−0.0007 (14)	0.0145 (14)	0.0006 (14)
Cl1	0.0704 (5)	0.0792 (6)	0.0717 (5)	−0.0083 (4)	0.0297 (4)	0.0167 (4)
N1	0.0432 (12)	0.0438 (14)	0.0372 (11)	−0.0062 (11)	0.0083 (9)	−0.0052 (10)
N2	0.0325 (11)	0.0582 (15)	0.0599 (14)	0.0049 (10)	−0.0067 (10)	−0.0175 (12)
O1	0.0559 (11)	0.0587 (13)	0.0631 (12)	−0.0200 (9)	0.0111 (9)	−0.0105 (10)
O2	0.0537 (11)	0.0443 (12)	0.0655 (12)	0.0071 (8)	0.0063 (9)	−0.0012 (9)
O3	0.0567 (10)	0.0718 (14)	0.0408 (11)	−0.0034 (9)	0.0059 (8)	0.0072 (10)
O4	0.0332 (8)	0.0530 (11)	0.0480 (10)	−0.0028 (7)	0.0028 (8)	−0.0075 (8)
O5	0.0436 (9)	0.0657 (13)	0.0483 (11)	−0.0010 (8)	0.0040 (8)	−0.0058 (9)

### Geometric parameters (Å, º)

**Table:**

C1—C2	1.384 (3)	C12—C13	1.376 (4)
C1—C6	1.386 (3)	C12—H12	0.9300
C1—N1	1.462 (3)	C13—C14	1.362 (4)
C2—C3	1.365 (3)	C13—H13	0.9300
C2—H2	0.9300	C14—C15	1.378 (3)
C3—C4	1.378 (4)	C14—H14	0.9300
C3—H3	0.9300	C15—Cl1	1.722 (3)
C4—C5	1.377 (4)	C16—O5	1.219 (3)
C4—H4	0.9300	C16—N2	1.365 (3)
C5—C6	1.381 (3)	C16—C17	1.496 (3)
C5—H5	0.9300	C17—C18	1.382 (3)
C6—C7	1.513 (3)	C17—C22	1.384 (3)
C7—C8	1.525 (3)	C18—C19	1.377 (4)
C7—H7A	0.9700	C18—H18	0.9300
C7—H7B	0.9700	C19—C20	1.374 (4)
C8—C9	1.483 (3)	C19—H19	0.9300
C8—H8A	0.9700	C20—C21	1.362 (4)
C8—H8B	0.9700	C20—H20	0.9300
C9—O3	1.190 (3)	C21—C22	1.386 (4)
C9—O4	1.391 (3)	C21—H21	0.9300
C10—C11	1.370 (4)	C22—H22	0.9300
C10—C15	1.388 (3)	N1—O2	1.221 (2)
C10—N2	1.425 (3)	N1—O1	1.230 (2)
C11—C12	1.380 (4)	N2—O4	1.417 (2)
C11—H11	0.9300		
			
C2—C1—C6	123.2 (2)	C13—C12—H12	120.0
C2—C1—N1	115.9 (2)	C11—C12—H12	120.0
C6—C1—N1	121.0 (2)	C14—C13—C12	120.1 (3)
C3—C2—C1	119.2 (2)	C14—C13—H13	119.9
C3—C2—H2	120.4	C12—C13—H13	119.9
C1—C2—H2	120.4	C13—C14—C15	120.1 (3)
C2—C3—C4	119.6 (3)	C13—C14—H14	119.9
C2—C3—H3	120.2	C15—C14—H14	119.9
C4—C3—H3	120.2	C14—C15—C10	120.3 (3)
C5—C4—C3	119.8 (3)	C14—C15—Cl1	119.4 (2)
C5—C4—H4	120.1	C10—C15—Cl1	120.4 (2)
C3—C4—H4	120.1	O5—C16—N2	120.1 (2)
C4—C5—C6	122.8 (2)	O5—C16—C17	122.9 (2)
C4—C5—H5	118.6	N2—C16—C17	117.0 (2)
C6—C5—H5	118.6	C18—C17—C22	119.0 (2)
C5—C6—C1	115.4 (2)	C18—C17—C16	118.0 (2)
C5—C6—C7	118.7 (2)	C22—C17—C16	123.0 (2)
C1—C6—C7	125.9 (2)	C19—C18—C17	120.4 (3)
C6—C7—C8	112.28 (19)	C19—C18—H18	119.8
C6—C7—H7A	109.1	C17—C18—H18	119.8
C8—C7—H7A	109.1	C20—C19—C18	120.0 (3)
C6—C7—H7B	109.1	C20—C19—H19	120.0
C8—C7—H7B	109.1	C18—C19—H19	120.0
H7A—C7—H7B	107.9	C21—C20—C19	120.3 (3)
C9—C8—C7	112.48 (19)	C21—C20—H20	119.9
C9—C8—H8A	109.1	C19—C20—H20	119.9
C7—C8—H8A	109.1	C20—C21—C22	120.3 (3)
C9—C8—H8B	109.1	C20—C21—H21	119.9
C7—C8—H8B	109.1	C22—C21—H21	119.9
H8A—C8—H8B	107.8	C17—C22—C21	120.0 (3)
O3—C9—O4	123.0 (2)	C17—C22—H22	120.0
O3—C9—C8	128.7 (2)	C21—C22—H22	120.0
O4—C9—C8	108.3 (2)	O2—N1—O1	123.2 (2)
C11—C10—C15	119.0 (2)	O2—N1—C1	119.14 (19)
C11—C10—N2	121.1 (2)	O1—N1—C1	117.7 (2)
C15—C10—N2	119.8 (2)	C16—N2—O4	118.59 (18)
C10—C11—C12	120.5 (3)	C16—N2—C10	126.6 (2)
C10—C11—H11	119.8	O4—N2—C10	114.47 (18)
C12—C11—H11	119.8	C9—O4—N2	114.17 (17)
C13—C12—C11	120.0 (3)		
			
C6—C1—C2—C3	2.3 (4)	N2—C16—C17—C18	131.7 (2)
N1—C1—C2—C3	−178.7 (2)	O5—C16—C17—C22	129.7 (3)
C1—C2—C3—C4	−1.4 (4)	N2—C16—C17—C22	−49.6 (3)
C2—C3—C4—C5	−0.4 (4)	C22—C17—C18—C19	2.1 (4)
C3—C4—C5—C6	1.4 (4)	C16—C17—C18—C19	−179.1 (2)
C4—C5—C6—C1	−0.5 (3)	C17—C18—C19—C20	−1.3 (4)
C4—C5—C6—C7	−179.5 (2)	C18—C19—C20—C21	−0.2 (4)
C2—C1—C6—C5	−1.4 (3)	C19—C20—C21—C22	1.1 (4)
N1—C1—C6—C5	179.72 (19)	C18—C17—C22—C21	−1.2 (4)
C2—C1—C6—C7	177.5 (2)	C16—C17—C22—C21	−179.9 (2)
N1—C1—C6—C7	−1.4 (3)	C20—C21—C22—C17	−0.4 (4)
C5—C6—C7—C8	−89.7 (3)	C2—C1—N1—O2	145.1 (2)
C1—C6—C7—C8	91.5 (3)	C6—C1—N1—O2	−35.9 (3)
C6—C7—C8—C9	161.9 (2)	C2—C1—N1—O1	−33.9 (3)
C7—C8—C9—O3	27.9 (4)	C6—C1—N1—O1	145.1 (2)
C7—C8—C9—O4	−150.2 (2)	O5—C16—N2—O4	169.69 (19)
C15—C10—C11—C12	0.5 (4)	C17—C16—N2—O4	−11.0 (3)
N2—C10—C11—C12	178.2 (2)	O5—C16—N2—C10	−3.3 (4)
C10—C11—C12—C13	1.5 (4)	C17—C16—N2—C10	176.0 (2)
C11—C12—C13—C14	−1.8 (4)	C11—C10—N2—C16	116.1 (3)
C12—C13—C14—C15	0.1 (4)	C15—C10—N2—C16	−66.3 (3)
C13—C14—C15—C10	1.9 (4)	C11—C10—N2—O4	−57.2 (3)
C13—C14—C15—Cl1	−177.1 (2)	C15—C10—N2—O4	120.5 (2)
C11—C10—C15—C14	−2.2 (4)	O3—C9—O4—N2	−7.8 (3)
N2—C10—C15—C14	−179.9 (2)	C8—C9—O4—N2	170.50 (18)
C11—C10—C15—Cl1	176.8 (2)	C16—N2—O4—C9	−67.4 (3)
N2—C10—C15—Cl1	−0.9 (3)	C10—N2—O4—C9	106.5 (2)
O5—C16—C17—C18	−49.0 (3)		

### Hydrogen-bond geometry (Å, º)

**Table:**

*D*—H···*A*	*D*—H	H···*A*	*D*···*A*	*D*—H···*A*
C8—H8*B*···Cl1^i^	0.97	2.68	3.627 (3)	167
C7—H7*A*···O1^ii^	0.97	2.60	3.520 (3)	158
C8—H8*A*···O2	0.97	2.47	3.092 (3)	121
C18—H18···O1^iii^	0.93	2.62	3.517 (4)	161
C4—H4···O1^iv^	0.93	2.64	3.468 (4)	149

Symmetry codes: (i) −*x*+2, −*y*+2, −*z*; (ii) −*x*+1, −*y*+1, −*z*; (iii) *x*+1/2, −*y*+3/2, *z*+1/2; (iv) *x*, *y*+1, *z*.
